# Deep Learning Models for Detection and Severity Assessment of Cercospora Leaf Spot (*Cercospora capsici*) in Chili Peppers Under Natural Conditions

**DOI:** 10.3390/plants14132011

**Published:** 2025-07-01

**Authors:** Douglas Vieira Leite, Alisson Vasconcelos de Brito, Gregorio Guirada Faccioli, Gustavo Haddad Souza Vieira

**Affiliations:** 1 EMEC, Sergipe Educational, Technology and Scientific Institute, Lourival Batista, Lourival Batista Highway s/n, Lagarto 49400-000, Sergipe, Brazil; 2 Laboratory of Embedded Systems and Robotics, Paraíba Federal University, Campus I Lot. Cidade Universitaria, João Pessoa 58051-900, Paraıba, Brazil; alisson@ci.ufpb.br; 3 Development and Environment Post-Graduation Program, Sergipe Federal University, Marcelo Déda Chagas Avenue, São Cristóvão 49107-230, Sergipe, Brazil; gregorioufs@gmail.com; 4 Postgraduate Program in Agroecology, Federal Institute of Espírito Santo (IFES), Santa Teresa Campus, ES-080 Highway, Km 93, São João de Petrópolis, Santa Teresa 29660-000, Espírito Santo, Brazil; ghsv@ifes.edu.br

**Keywords:** convolutional neural networks, CNN, plant disease severity, cercospora leaf spot

## Abstract

The accurate assessment of plant disease severity is crucial for effective crop management. Deep learning, especially via CNNs, is widely used for image segmentation in plant lesion detection, but accurately assessing disease severity across varied environmental conditions remains challenging. This study evaluates eight deep learning models for detecting and quantifying Cercospora leaf spot (*Cercospora capsici*) severity in chili peppers under natural field conditions. A custom dataset of 1645 chili pepper leaf images, collected from a Brazilian plantation and annotated with 6282 lesions, was developed for real-world robustness, reflecting real-world variability in lighting and background. First, an algorithm was developed to process raw images, applying ROI selection and background removal. Then, four YOLOv8 and four Mask R-CNN models were fine-tuned for pixel-level segmentation and severity classification, comparing one-stage and two-stage models to offer practical insights for agricultural applications. In pixel-level segmentation on the test dataset, Mask R-CNN achieved superior precision with a Mean Intersection over Union (MIoU) of 0.860 and F1-score of 0.924 for the mask_rcnn_R101_FPN_3x model, compared to 0.808 and 0.893 for the YOLOv8s-Seg model. However, in severity classification, Mask R-CNN underestimated higher severity levels, with an accuracy of 72.3% for level III, while YOLOv8 attained 91.4%. Additionally, YOLOv8 demonstrated greater efficiency, with an inference time of 27 ms versus 89 ms for Mask R-CNN. While Mask R-CNN excels in segmentation accuracy, YOLOv8 offers a compelling balance of speed and reliable severity classification, making it suitable for real-time plant disease assessment in agricultural applications.

## 1. Introduction

Plant diseases pose a significant threat to global agriculture, causing substantial economic losses estimated at USD 220 billion annually and affecting crop yield, quality, and food security [1,2]. The accurate assessment of plant disease severity is critical for effective crop management, predicting yield losses, and implementing targeted disease control measures. Moreover, it helps minimize unnecessary chemical applications, reducing health and environmental risks [3,4].

In Brazil, chili peppers, particularly Capsicum species, hold significant economic and cultural importance due to their culinary uses, nutritional properties, and role in family agriculture [5]. These peppers exhibit diverse biochemical compositions, including capsaicinoids, phenolic compounds, and antioxidants, which vary based on species, ripeness, and harvest year [6,7]. However, pepper cultivation faces challenges, notably pesticide overuse [5]. One major disease affecting Brazilian chili peppers is Cercospora leaf spot (*Cercospora capsici*), a destructive fungal disease that also impacts crops like sugar beet, roses, and wheat [8,9]. This disease causes circular lesions on leaves, leading to defoliation and significant yield losses. Consequently, the precise assessment of its severity is essential for effective management [9].

Traditional digital image processing techniques have facilitated the development of methods to assess plant disease severity. These methods involve capturing images of affected plants and computationally analyzing visual characteristics of lesions or symptoms [10]. Software tools such as ImageJ [11], Assess2, and Fiji [12] are commonly used to manipulate images and quantify leaf areas and lesions. However, these tools often require human intervention to identify lesion colors, which can lead to confusion with non-disease lesions and underestimation of severity due to the misinterpretation of hues representing diseased tissue [13].

In addition to methods based on RGB image analysis, some approaches also make use of multispectral camera analysis for plant disease detection. While RGB imaging captures visible light in three bands (red, green, and blue) and is known for its accessibility and low cost, other strategies incorporate data from different wavelength bands, including regions beyond the visible spectrum such as near-infrared and red-edge [14]. In these cases, spectral indices such as the Normalized Difference Red-edge Index (NDRE) and the Normalized Difference Vegetation Index (NDVI) are commonly used to monitor physiological indicators of plant stress, offering complementary information to that provided by RGB imagery [14,15].

Automated sensor-based technologies, including image processing and machine learning, particularly Convolutional Neural Networks (CNNs), are increasingly utilized to enhance accuracy and efficiency [16]. CNNs have proven highly effective in plant disease detection, excelling in feature extraction and classification across numerous studies [17,18]. Over time, CNN-based models have achieved significant advancements in image classification, object detection, and instance segmentation, making them valuable tools for precision agriculture [19].

Recent advances in AI-driven agriculture emphasize the importance of transfer learning, model interpretability, and robust architectures to enhance generalization and performance in real-world scenarios [20,21,22]. Within image-based applications, instance segmentation—a computer vision task that combines object detection and semantic segmentation—plays a key role by detecting, classifying, and delineating individual object instances in an image with pixel-level precision [23,24]. This deep learning approach has become essential in precision agriculture, enabling accurate identification and localization of crops, weeds, and disease symptoms across diverse crop types, while also supporting detailed severity assessment to guide more effective agricultural management strategies [25].

Two architectures stand out in instance segmentation for agriculture: Mask R-CNN and YOLO (You Only Look Once). Mask R-CNN has shown promising results in detecting and segmenting plant diseases, including paddy crop diseases [26], apple leaf rust [27], wheat powdery mildew [28], cotton leaf diseases [29], and weeds [30]. Similarly, YOLOv8 models have been applied to leaf disease detection and segmentation in crops such as tomatoes [31], rice [32], and wheat [24], achieving precision and recall rates above 95% in many cases [33,34].

Mask R-CNN and YOLOv8 were chosen for their complementary strengths in instance segmentation, essential for lesion detection and severity assessment. Mask R-CNN’s two-stage architecture, leveraging a region proposal network and feature pyramid network, delivers high precision for detailed segmentation. In contrast, YOLOv8’s one-stage approach prioritizes speed, ideal for real-time agricultural applications. Given their distinct inference styles and the absence of specialized models for Cercospora leaf spot in chili peppers, these models provide a robust baseline for evaluating precision versus efficiency in real-world conditions.

Comparative studies of these frameworks reveal mixed results. Some demonstrate YOLOv8’s superiority over Mask R-CNN in accuracy and inference speed, particularly in orchard environments [35]. Conversely, other studies indicate that Mask R-CNN performs comparably or better in specific scenarios [36,37,38]. These findings highlight the need for context-specific evaluations.

Deep learning for crop disease detection and severity assessment faces challenges such as limited generalization across diverse environmental conditions, high computational costs, and data scarcity for specific crops [39,40]. While YOLO and Mask R-CNN have advanced instance segmentation, their performance varies in natural settings with variable lighting and backgrounds, and few studies focus on custom models for chili peppers, with most targeting crops like sugar beet [41,42]. The scarcity of custom datasets and comparative studies further hinders robust model development [35]. This study addresses these gaps by systematically evaluating custom YOLOv8 and Mask R-CNN models on a novel dataset tailored for Cercospora leaf spot severity assessment in chili peppers.

Based on the literature review and identified gaps, this study tests the hypothesis that instance segmentation models with different architecture approaches exhibit significant differences in accuracy, speed, and robustness when assessing Cercospora severity in chili peppers under natural conditions. The main objective is to systematically compare YOLOv8 and Mask R-CNN architectures using a custom dataset of 1645 chili pepper leaf images with 6282 annotated lesions, to determine which approach offers the best balance between segmentation accuracy and computational efficiency for practical disease severity assessment in real-world agricultural settings.

### 1.1. YOLOv8

YOLO [43], provided by Ultralytics, is a one-stage detection framework that enables object localization, classification, and segmentation in a single pass through the network, effectively balancing speed and precision [44]. Originally designed for object detection, YOLO algorithms have evolved significantly, and since 2023, YOLOv8-seg models have expanded to include segmentation tasks by leveraging principles from the YOLACT network [45], enabling pixel-level instance segmentation alongside traditional detection capabilities.

The architecture of YOLOv8-Seg is built around several key components, starting with the backbone, which extracts intricate features from input images. This is complemented by the C2f (Context Fusion and Feature Fusion) module, which integrates contextual information from diverse layers and scales to enhance object recognition in complex scenes, and the SPPF (Spatial Pyramid Pooling Fast) module, which preserves spatial resolution while capturing multi-scale contextual information to improve detection robustness. The neck module, incorporating Feature Pyramid Networks (FPNs), bridges the backbone and the head, enriching feature maps across different scales to ensure accurate predictions. The head of YOLOv8-Seg generates final predictions, including bounding boxes, object classes, and segmentation masks, using anchor boxes and grid cells, while a single-shot module produces prototype masks that are refined for precise segmentation and enhanced object boundary delineation. Notably, YOLOv8-Seg maintains YOLO’s fundamental architecture for object detection while introducing an additional output module in the head to generate mask coefficients, seamlessly integrating segmentation capabilities into the framework.

**Figure 1:**
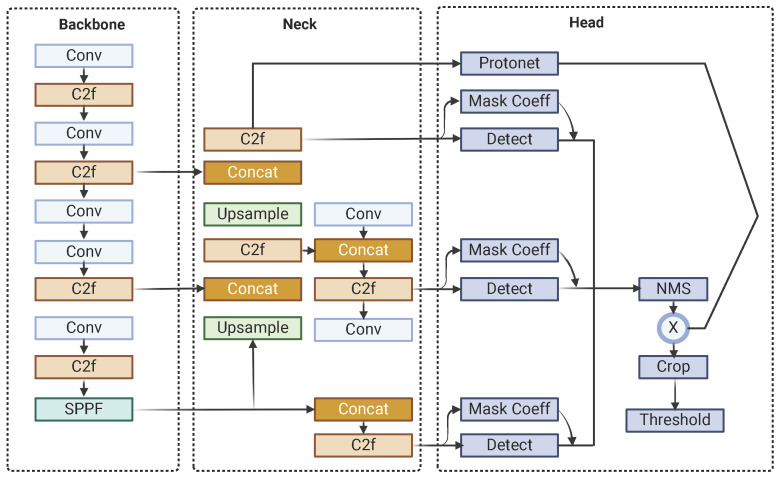
YOLOv8-Seg architecture.

Post-processing steps further refine the model’s outputs to ensure high-quality results. These steps include Non-Maximum Suppression (NMS) to eliminate duplicate detections and thresholding to filter out low-confidence predictions, ensuring that the final outputs are both accurate and reliable for real-world applications. Figure 1 shows the YOLOv8-Seg architecture.

### 1.2. Mask R-CNN

Released by Facebook AI Research in 2019 [46] as part of the Detectron2 framework, Mask R-CNN is a deep learning model renowned for its versatility and effectiveness in various computer vision tasks [47]. Built on the PyTorch v2.6 deep learning library, Mask R-CNN follows a two-stage approach and features four main components: the backbone, the neck, the region proposal network (RPN), and the head.

In the first stage, the backbone and the neck work together to extract and process features from the input image. The backbone is responsible for feature extraction, utilizing various pre-trained architectures like ResNet. These architectures, trained on large image datasets such as ImageNet, enable the backbone to learn rich feature representations crucial for downstream tasks. The backbone network includes several convolutional layers organized hierarchically, capturing multiple-level features. The neck component is implemented as an FPN, which enhances the backbone’s capabilities by creating a multi-scale feature pyramid. The FPN combines low-resolution, semantically robust features with high-resolution, semantically weak features through a top–down pathway and lateral connections, allowing the network to generate robust feature maps at various scales.

**Figure 2:**
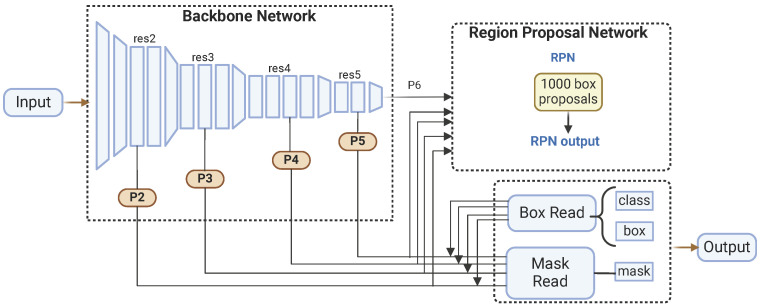
Mask R-CNN architecture.

In the second stage, the region proposal network (RPN) and the head refine these feature maps to produce final outputs. The RPN generates region proposals based on the feature maps from the FPN, identifying approximately 1000 box proposals likely to contain objects. This is achieved by sliding a small network over the feature maps and predicting each anchor point’s objectness score and bounding box coordinates. The RPN efficiently narrows down the regions of interest. Subsequently, the head component refines these region proposals and produces segmentation masks. It performs further classification and regression to fine-tune the bounding boxes and predicts binary masks for each detected object. Figure 2 shows the Mask R-CNN architecture.

## 2. Materials and Methods

**Figure 3:**
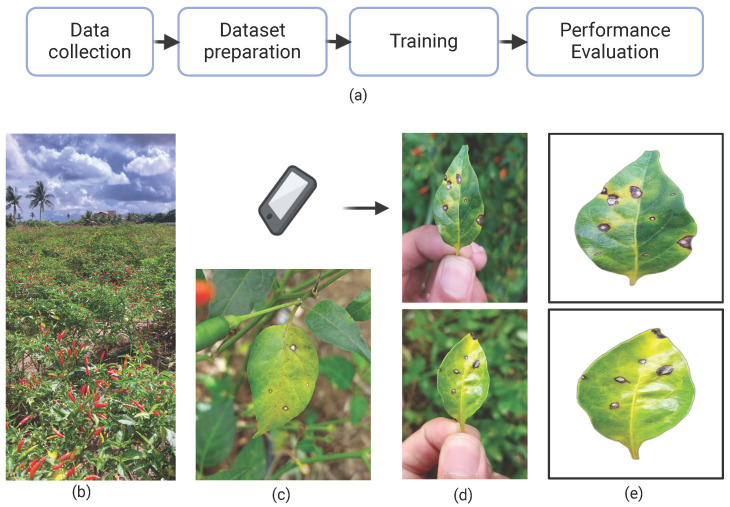
(**a**) Overall research workflow; (**b**) plantation where samples were taken; (**c**) leaf affected by Cercospora leaf spot; (**d**) examples of sample collections; and (**e**) samples after preprocessing.

**Figure 4:**
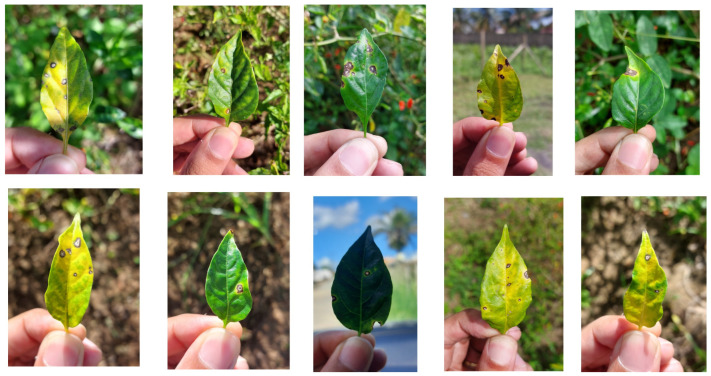
Sample of chili pepper leaves collected for the dataset.

This study comprised four main stages: data collection, dataset preparation, model training, and performance evaluation, as illustrated in Figure 3a. Initially, RGB images of chili pepper leaves were captured in the field (Figure 4), including both healthy leaves and those affected by Cercospora leaf spot (Figure 3c). The images were taken under natural conditions, with varying environmental and lighting settings over different days. After preprocessing, the images were manually annotated to generate the training and test datasets. The annotated training data were then used to train eight models based on the Mask R-CNN and YOLOv8-Seg architectures. Finally, the performance of the trained models was evaluated using the test dataset.

### 2.1. Dataset Acquisition

The dataset comprises 1645 images of chili pepper leaves collected between 15 March and 31 May 2023, in a plantation located in Lagarto, Sergipe State, Brazil (Lat: −10.9145, Long: −37.6639), as shown in Figure 3b. Image acquisition was performed using the main rear camera of a Samsung Galaxy A52 smartphone. The sample size was defined based on trends observed in similar studies involving custom datasets for plant disease detection. These studies typically used between 1000 and 2000 images to train models effectively for tasks such as lesion segmentation and classification, achieving robust performance outcomes [48,49,50,51].

The camera specifications include a primary sensor with a 64 MP resolution, an aperture of f/1.8, a focal length of approximately 26 mm (35 mm equivalent), a pixel size of approximately 0.8 µm, phase detection autofocus (PDAF), and optical image stabilization (OIS). The resulting images were in JPEG format (RGB mode) with dimensions of 4624 pixels (width) × 2084 pixels (height), yielding a total resolution of 9.64 MP and a resolution of 72.0 DPI (horizontal) × 72.0 DPI (vertical). Typical EXIF metadata extracted from the images indicate an aperture of f/1.8, an exposure time of approximately 0.002079 s, and an ISO setting of 25.

The images were captured in a plantation under natural lighting and environmental conditions, where a “natural environment” refers to outdoor lighting and varying backgrounds, as opposed to a controlled environment with regulated lighting and homogeneous backgrounds. To maintain consistency in image acquisition, an approximate camera-to-object distance of 10 cm was maintained whenever possible. This distance was chosen to ensure that the leaves filled the frame adequately, leveraging the camera’s 26 mm focal length and PDAF for sharp focus, while minimizing variations in perspective and scale.

To optimize the process, affected leaves were detached and photographed, as illustrated in Figure 3d and Figure 4. This approach simplified image acquisition, as in situ photography is often labor-intensive due to challenges in isolating leaves on the plant and avoiding occlusion by other plant parts. In potential mobile applications using these models—where assessing average crop severity may involve analyzing dozens of leaves—removing the leaves significantly improves efficiency. As the preprocessing step effectively removes the background, the difference between in situ and detached-leaf images becomes negligible. Consequently, key environmental characteristics—such as lighting variation and background heterogeneity—are preserved in the raw images. Figure 4 shows a sample of the collected leaves, illustrating differences in background, lighting, shadow patterns, lesion features, and leaf coloration.

### 2.2. Dataset Preparation

The images were preprocessed before training to standardize the samples using a Python v3.14 script with the OpenCV library. The script processes raw images by applying a crop operation to define a region of interest (ROI), producing a zoom effect, and then resizing them to 640 × 640 pixels. The Rembg library [52] was used for automatic background removal. This preprocessing step, involving resizing and background removal, may introduce minor compression artifacts, as observed in the processed samples (Figure 3e). The dataset is available online under the following DOI: 10.5281/zenodo.13272038.

All 6282 lesions identified in the 1645 field-captured leaf images were manually annotated using the Roboflow API tool, following the COCO dataset specifications. This annotation process was carried out with the assistance of specialists for accurate lesion identification. The annotated samples were then randomly divided into training, validation, and test datasets, as detailed in Table 1.

### 2.3. Models and Training

Four pre-trained YOLOv8-Seg models were tested: YOLOv8n-Seg (nano), YOLOv8s-Seg (small), YOLOv8m-Seg (medium), and YOLOv8l-Seg (large). These models differ in their response speed, segmentation capacity, and accuracy.

Additionally, four Mask R-CNN models were utilized. These include mask_rcnn_R101_3x and mask_rcnn_R50_3x. The mask_rcnn_R101_3x model employs a ResNet-101 backbone with 101 layers, while the mask_rcnn_R50_3x model uses a ResNet-50 backbone with 50 layers. Both models incorporate an FPN for improved multi-scale feature extraction. Finally, mask_rcnn_R101_C4_3x and mask_rcnn_R50_C4_3x models were tested. These models also use ResNet-101 and ResNet-50 as their backbones but differ from the FPN-based models by utilizing the C4 (Convolutional Cascade for Object Detection) block.

The pre-trained models were used as a starting point for training the custom dataset. YoloV8 and Mask-RCNN employ different training procedures: YoloV8 utilizes epochs, whereas Detectron2 relies on the number of iterations. The recommended 300 epochs suggested by Ultralytics were adhered to as a reference. The corresponding number of iterations for Mask-RCNN was then calculated based on the train dataset’s sample size (1040) and a batch size of 16, resulting in 19,500 iterations. The learning rate was set at 0.001. All models were trained and tested using a Google Colab environment, equipped with an NVIDIA Tesla T4 GPU supported by CUDA version 12.2 and up to 15 GB of RAM.

The quality of the training were evaluated through the mAP50 metric applied in the segmentation task to the valid dataset. The mAP50 calculates the mean of the average precision values for all classes, where precision is assessed at an IoU of 0.5. The mAP50 can be mathematically expressed as follows:

(1) $${\mathrm{mAP}}_{50}=\frac{1}{N}\sum_{i=1}^{N}{\mathrm{AP}}_{50}\left(i\right)$$

where *N* is the total number of classes, and ${\mathrm{AP}}_{50}\left(i\right)$ represents the average precision for class *i* at an IoU threshold of 0.5. The average precision (${\mathrm{AP}}_{50}\left(i\right)$) for a specific class is calculated as the area under the precision–recall curve for that class, given by

(2) $${\mathrm{AP}}_{50}\left(i\right)=\int_{0}^{1}p_{50}\left(r\right)\;dr$$

where $p_{50}\left(r\right)$ denotes the precision at a recall level *r* for IoU = 0.5.

### 2.4. Inference Confidence Threshold (CT)

The confidence threshold (CT) is a crucial parameter for filtering object detections and segmentation results in both YOLOv8 and Mask R-CNN frameworks [53]. It plays a pivotal role in determining the validity of predicted segments or masks. Both models assign confidence scores to each predicted pixel or segment, indicating the likelihood of it belonging to a particular class. For example, a confidence score of 0.9 indicates a 90% certainty in the segmentation’s correctness. The CT can be adjusted during inference to influence the outcomes, allowing for fine-tuning of the model’s performance. In this study, all evaluated metrics were analyzed by varying the CT from 0.1 to 0.9 in 0.1 increments.

### 2.5. Severity Determination

The severity of a disease in a plant can be considered as the proportion of the plant unit showing visible disease symptoms, usually expressed as a percentage, according to the following equation [54]:

(3) $$S=\frac{Area\;of\;diseased\;leaf\;tissue}{Total\;leaf\;area}\times 100\%$$

An algorithm was developed to determine the ground truth lesion areas. It accesses the annotations previously created in COCO format using the Roboflow API. It generates a normalized binary mask where white pixels (value = 1) represent the lesion, and black pixels (value = 0) indicate no lesions. The algorithm then counts the number of white pixels to reveal the damaged area. Similarly, the code accesses the models’ predicted masks and creates binary masks for the predicted lesion areas, performing the same procedure for calculating the lesioned area used in the ground truth masks.

To calculate the total area of the leaf, the preprocessed image undergoes a contour detection algorithm, utilizing methods such as Otsu Thresholding and Dilate. The process begins with converting the preprocessed image to grayscale and applying Otsu’s method for thresholding. Subsequently, a dilate operation is applied to accentuate the contours, followed by the identification of these contours in the processed image. Finally, the total area in pixels inside the contour is determined.

### 2.6. Severity-Level Classification

**Figure 5:**
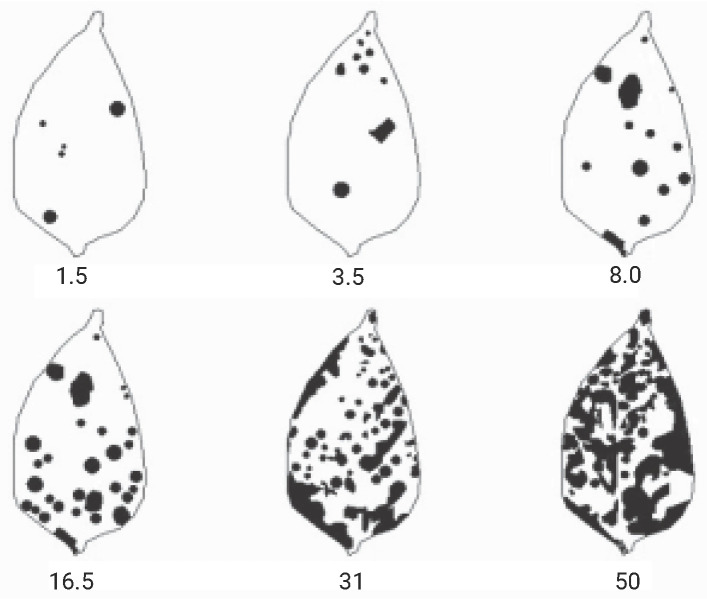
Diagrammatic scale for assessing Cercospora leaf spot in bell pepper (*Capsicum annuum*), indicating severity levels of 1.5%, 3.5%, 8.0%, 16.5%, 31.0%, and 50.0%. Adapted from [55].

Diagrammatic scales were used as references for classifying the levels of disease severity. Given the lack of specific scales for the Cercospora leaf spots on *Capsicum frutescens* (chili pepper) in the literature, the severity scale developed by the authors of [55] for *Capsicum annuum* (bell pepper) was utilized (Figure 5). This choice was justified by the fact that Cercospora leaf spot affects both plants and they belong to the same family, *Solanaceae*. Based on Michereff’s scale, the severity was categorized into seven distinct classes, as shown in Table 2.

### 2.7. Evaluation Metrics

In this phase, the custom-trained models were evaluated using images from the test dataset that were unseen during the model training. The assessment involved applying metrics at both the pixel level and in the final classification of severity levels.

#### 2.7.1. Pixel-Level Evaluation Metrics

The ground truth and predicted lesion masks were compared using pixel-by-pixel evaluation metrics to determine accuracy, precision, recall, and F1-score, considering the True Positive (TP), True Negative (TN), False Positive (FP), and False Negative (FN) pixels.

TP refers to a pixel correctly identified as part of the lesion in the predicted and ground truth masks. TN denotes a pixel accurately recognized as not part of the lesion in both masks. FP describes a pixel incorrectly identified as part of the lesion in the predicted mask when it is not part of the lesion in the ground truth mask. FN indicates a pixel wrongly identified as not part of the lesion in the predicted mask while it is part of the lesion in the ground truth mask.

According to [56], Accuracy (Equation (4)) is a commonly used performance metric that measures the model’s overall correctness in making predictions. It is the ratio of correctly predicted samples to the total number of samples in the dataset. Precision (Equation (5)) is a measure that indicates the proportion of correct positive predictions out of the total positive predictions made by the model. It is calculated by dividing the number of TPs by the sum of TPs and FPs. Recall (Equation (6)), or sensitivity, is the proportion of lesions truly detected by the model out of the total number of existing lesions. F1-score (Equation (7)) is a measure that combines precision and recall into a single metric to provide a balanced view of model performance. It is the harmonic mean between precision and recall.

(4) $$Accuracy=\frac{TP+TN}{TP+TN+FP+FN}\times 100\%$$

(5) $$Precision=\frac{TP}{TP+FP}\times 100\%$$

(6) $$Recall=\frac{TP}{TP+FN}\times 100\%$$

(7) $$F1-score=\frac{2\times precision\times recall}{precision+recall}\times 100\%$$

In addition to the metrics, the average inference times of all models on the test dataset were recorded.

The Mean Intersection Over Union (MIoU) (Equation (8)) metric was used to assess the segmentation quality of the models. This metric evaluates performance by considering the overlap and union areas between the ground truth and predicted masks.

(8) $$\mathrm{MIoU}=\frac{1}{N}\sum_{i=1}^{N}\frac{TP_{i}}{TP_{i}+FP_{i}+FN_{i}}=\frac{1}{N}\sum_{i=1}^{N}\frac{\mathrm{Area}\;\mathrm{of}\;{\mathrm{overlap}}_{i}}{\mathrm{Area}\;\mathrm{of}\;{\mathrm{union}}_{i}}$$

#### 2.7.2. Severity-Level Evaluation Metrics

The ground truth and predicted severity values were evaluated using linear regression, the coefficient of determination ($R^{2}$), and Root Mean Square Error (RMSE) (Equation (9)). A KDE (Kernel Density Estimation) plot was created to analyze the residuals, and a boxplot was used to evaluate the deviations by severity level. Following this, the ground truth and predicted severity levels of all models were compared using confusion matrices. Finally, the accuracy, precision, recall, and F1-score for severity-level classification were computed:

(9) $$RMSE=\sqrt{\frac{1}{n}\sum_{i=1}^{n}{\left(Y_{i}-\hat{Y_{i}}\right)}^{2}}$$

**Figure 6:**
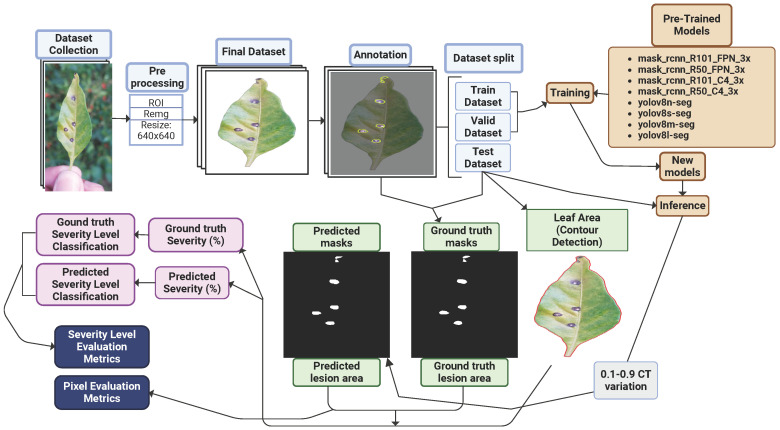
Diagram of the method’s steps.

The diagram in Figure 6 illustrates the detailed methodology procedure.

## 3. Results

The mAP50 scores of the models (Table 3) obtained from the validation dataset demonstrated the success of the training and the defined hyperparameters, ranging from a minimum value of 0.893 for the mask_rcnn_R50_FPN_3x model to a maximum of 0.925 for the YOLOv8n-Seg model. These values indicate the robustness of the models, which were then used to evaluate the metrics on the test dataset, as will be discussed in the following sections.

### 3.1. Pixel-Level Evaluation

**Figure 7:**
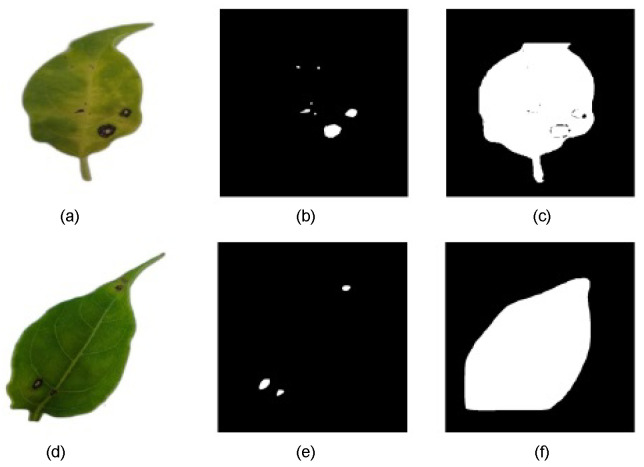
(**a**) RGB-preprocessed leaf image sample. (**b**) Binary-predicted lesion mask with mask_rcnn_R101_3x model at CT = 0.6. (**c**) Binary-predicted lesion mask with mask_rcnn_R101_3x model at CT = 0.1. (**d**) RGB-preprocessed leaf image sample. (**e**) Binary-predicted lesion mask with YOLOv8s-Seg model at CT = 0.8. (**f**) Binary-predicted lesion mask with YOLOv8s-Seg model at CT = 0.2.

The results indicate that some models struggled to accurately segment lesions at low CTs, often misclassifying large portions of the leaf surface as lesions. Figure 7 shows two binary predicted mask examples in different CTs. To ensure a precise pixel-level evaluation and prevent result distortion, data from CT levels where significant errors occurred were excluded. As a result, some subsequent graphs may show discontinuities at lower CT levels.

**Figure 8:**
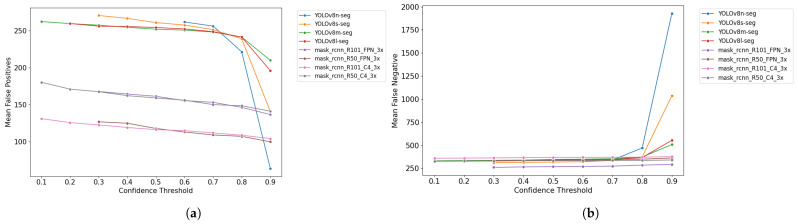
(**a**) Mean False Positives vs. confidence threshold. (**b**) Mean False Negatives vs. confidence threshold.

The graphs in Figure 8 display the distribution of the means of FPs and FNs across the test dataset at different CT levels, ranging from 0.1 to 0.9.

The data reveal a generally expected trend for all models: as CT increases, FPs tend to decrease, while FNs increase. As the CT is raised, models become more selective. It means that detections made with lower confidence, often associated with FPs, are discarded, reducing their occurrence. However, this increased selectivity makes models more likely to reject true detections, increasing FNs.

The graphs also indicate that Mask R-CNN-based models tend to exhibit lower values for FPs and FNs than YOLOv8-based models, with the most significant difference observed in FPs. Subsequent sections will explore the impact of this behavior on other results. Additionally, the graphs highlight a more significant imbalance in YOLOv8-based models at higher CT levels.

These findings are reflected in the computation of pixel-level metrics. Table 4 presents the overall averages of these metrics, the CT value or range for the best results, and the average inference time for each model on the test dataset.

The MIoU results indicate that the overlap between ground truth and predicted masks exceeded 80% for all models. All Mask R-CNN-based models achieved higher values than the YOLOv8 models, with mask_rcnn_R101_FPN_3x standing out by achieving the highest MIoU value among the tested models, at 86%.

It is crucial to note that pixel accuracy is not a suitable comparative metric in this context. Due to the nature of the images, the number of TNs for binarized images significantly outweighs the number of TPs, leading to an imbalance and resulting in artificially high accuracy values (99.9% for Mask R-CNN and 99.8% for YOLOv8-based models).

Precision, recall, and F1-scores also demonstrate that Mask R-CNN-based models generally achieve better results in pixel-level metrics. All the Mask R-CNN models achieved higher F1-scores. On the other hand, the average inference times of the YOLOv8-based models were significantly lower. The mask_rcnn_R50_C4_3x model had the highest average inference time, at 477 ms, while the YOLOv8n-Seg model had the lowest, at 27 ms.

### 3.2. Severity-Level Evaluation

The automatic calculation of ground truth severity in the test dataset of 309 samples identified 46 healthy samples, 133 level I samples, 105 level II samples, and 25 level III samples. This means that from the field-collected samples, the highest severity observed did not exceed 8% of the leaf area affected by the disease.

Table 5 presents the best RMSE values (comparing ground truth and predicted severities) along with their corresponding CTs.

The mask_rcnn_R101_FPN_3x model achieved the lowest RMSE of 0.132, while among YOLOv8-based models, YOLOv8s-Seg performed best with an RMSE of 0.136. In the RMSE analysis across confidence thresholds (CTs), the best RMSE values occurred at lower CT levels, specifically at each model’s minimum CT. To explore this, the sum of predicted and ground truth lesion pixel areas was assessed for each CT value. Regarding the data range, the RMSE represents less than 0.2% of the total scale, indicating a small error overall. However, the impact is more noticeable in lower severity categories like level I (0–1.5%), where the RMSE of 0.132 represents 8.8% of the interval.

**Figure 9:**
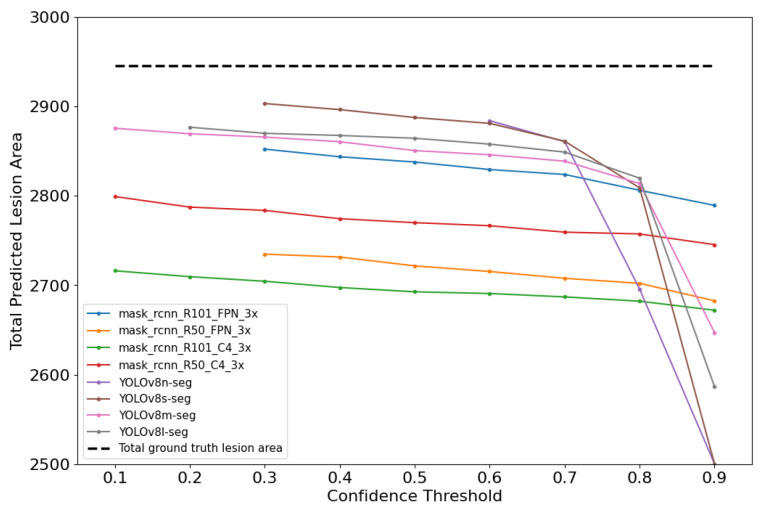
Total predicted and ground truth lesion area for each model evaluation.

As illustrated in Figure 9, it was observed that the gap between the predicted and ground truth areas (dashed line) widened as the CT increased. At lower CTs, the predicted areas were more closely aligned with the ground truth areas.

Lower CTs allow for a more comprehensive detection by including a wider range of predictions, while higher CTs are more restrictive and result in fewer detections, as reflected in the analysis of False Positives (FPs) and False Negatives (FNs). Both frameworks showed a tendency to predict smaller lesion areas compared to the ground truth; however, this was more pronounced in Mask R-CNN-based models, which generally produced smaller predicted areas than YOLOv8-based models. This suggests that, although Mask R-CNN achieves higher pixel-level precision by reducing FPs, its conservative inference leads to an underestimation of lesion size. Interestingly, at lower CTs—where FPs naturally increase—the enlargement of predicted masks partially offset this underestimation bias, resulting in lower and best RMSE values.

After performing inferences using the eight models on the test dataset and classifying the severity levels, the predicted severity-level classifications were compared with the ground truths using confusion matrices. From these matrices, precision, recall, and F1-score for severity classification were calculated. Table 6 shows the results.

The classification metrics for all models exceeded 90%. Among them, the YOLOv8n-Seg model achieved the highest classification precision at 95.2%, while the Mask R-CNN model with the best performance was mask_rcnn_R101_FPN_3x, with 94.9%. Overall, YOLOv8-based models outperformed Mask R-CNN-based models in classification. Despite having a better RMSE, the mask_rcnn_R101_FPN_3x model performed slightly worse than YOLOv8n-Seg in classification precision. This discrepancy arises because severity-level classifications are made in intervals and do not necessarily reflect the continuous errors represented by RMSE.

**Figure 10:**
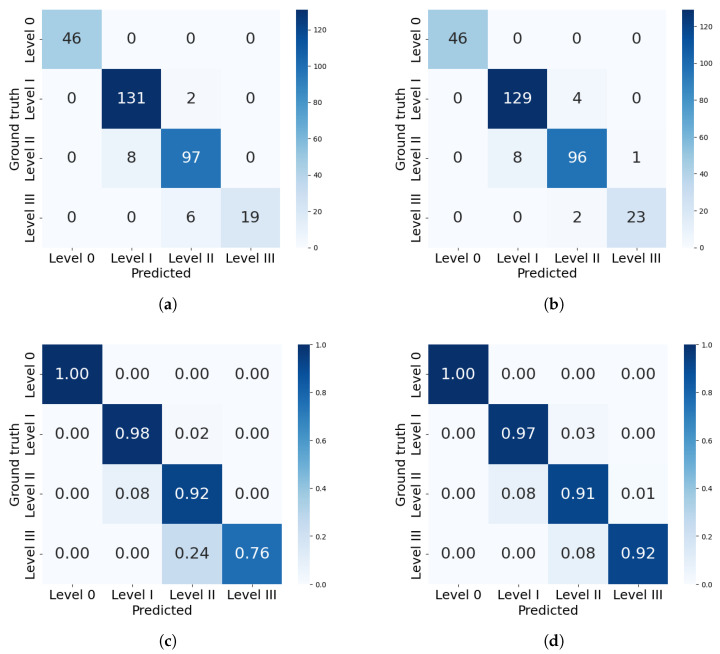
Confusion matrices for severity classification by the best models from each framework. (**a**) Confusion matrix for mask_rcnn_R101_FPN_3x. (**b**) Confusion matrix for YOLOv8n-Seg model. (**c**) Percentual confusion matrix for mask_rcnn_R101_FPN_3x. (**d**) Percentual confusion matrix for YOLOv8n-Seg model.

The analysis will now focus more on the classification results of the best models from each framework: mask_rcnn_R101_FPN_3x and YOLOv8n-Seg. Figure 10 presents the confusion matrices with the best performances in severity classification for each model. The confusion matrix includes severity levels 0 to III, as these were the only levels present in the test dataset.

Both models correctly identified all healthy leaves (level 0), achieving 100% precision in this category. The mask_rcnn_R101_FPN_3x model tends to underestimate severity. As severity increases, classification errors become more pronounced: 8% of level II leaves were misclassified as level I, and 24% of level III leaves were misclassified as level II. Similarly, the YOLOv8n-Seg model also underestimates levels II and III, but with a lower deviation of 8% in both cases. This difference is reflected in the precision per level, where Mask R-CNN’s performance declined to 72.3% for severity level III, while YOLO achieved 91.4%.

**Figure 11:**
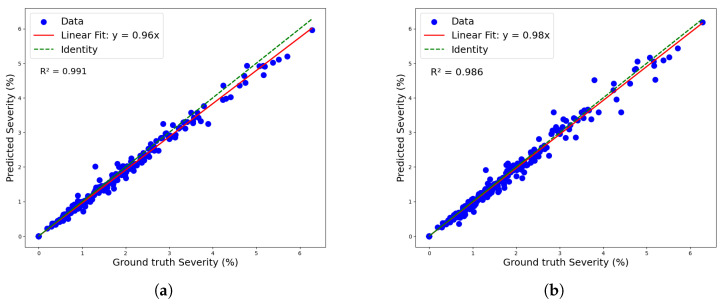
Linear regression for severity classification: (**a**) maskrcnn_R101_FPN_3x model and (**b**) YOLOv8n-Seg model.

**Figure 12:**
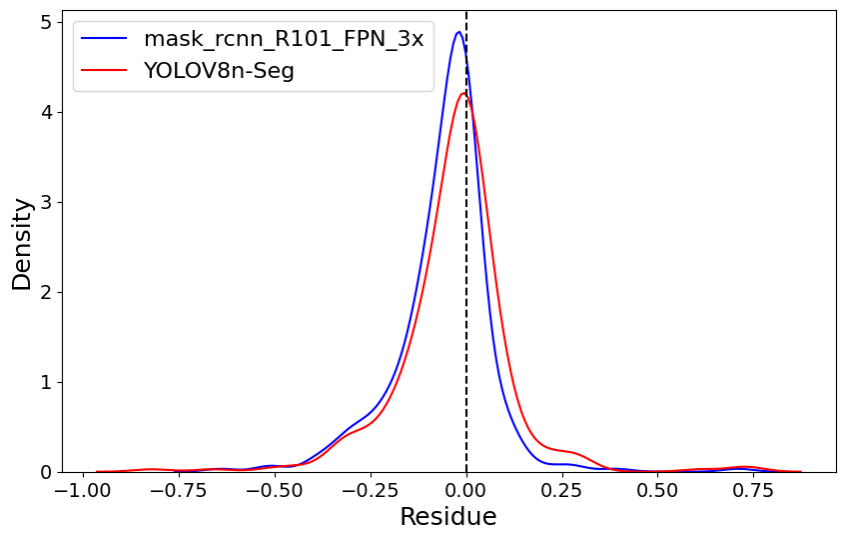
Kernel Density Estimation (KDE) plot.

Figure 11 shows both models’ linear regressions between predicted and reference severity. As indicated by the previously mentioned RMSE values, the coefficients of determination also highlight a superior data fit by the mask_rcnn_R101_FPN_3x model compared to the YOLOv8n-Seg model, with R^2^ values of 0.991 and 0.986, respectively. However, the deviation of the regression line from the identity line confirms a greater tendency for underestimation by the mask_rcnn_R101_FPN_3x model. This tendency is further illustrated by the Kernel Density Estimation (KDE) plot for the residuals (predicted-observed) of both models in Figure 12.

Both models exhibit left-skewed curves with a predominance of negative residuals. However, the mask_rcnn_R101_FPN_3x model shows a more pronounced skew and a higher peak, indicating a greater number of negative residuals.

To evaluate the behavior of residuals by severity level, boxplot graphs were created for each model at levels I, II, and III. These graphs compare the distribution of residuals between the reference severity values and the predicted values for the three different severity levels representing diseased leaves.

**Figure 13:**
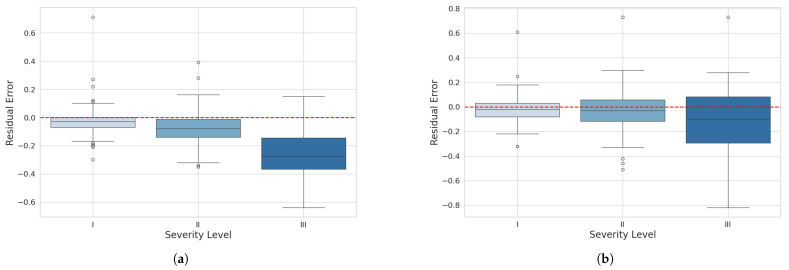
Residual boxplot for severity classification: (**a**) maskrcnn_R101_FPN_3x model and (**b**) YOLOv8n-Seg model.

In the boxplot for the mask_rcnn_FPN_3x model (Figure 13a), at severity level I, most of the residuals are centered around zero with a relatively symmetric distribution. At severity level II, the residual distribution is narrower, centered around zero, with few outliers and a slight tendency for underestimation. At severity level III, the distribution is wider and shifted toward negative values, indicating a tendency to underestimate higher severities with increased variability.

For the YOLOv8n-Seg model, at severity level I, most residuals are also centered around zero, but with fewer outliers and a slightly narrower interquartile range, suggesting lower variability. At severity level II, the residual distribution is comparable to the mask_rcnn_R101_FPN_3x model, centered around zero with few outliers and a slightly wider interquartile range, indicating slightly higher variability. At severity level III, the residual distribution shows a tendency for underestimation, but with less variability and a moderate amount of outliers, with some residuals extending into more negative values.

In conclusion, both plots indicate that the models tend to underestimate higher severities, but the YOLOv8n-Seg model demonstrates greater consistency and lower prediction variability than the mask_rcnn_R101_FPN_3x. This suggests that the YOLOv8 model performs slightly better in precision and consistently predicting severity levels.

## 4. Discussion

This study demonstrated the efficacy of both Mask R-CNN and YOLOv8-based models in the automatic lesion identification and severity level classification of Cercospora leaf spot in chili pepper leaves. The distinct inference natures of these frameworks (one-stage for YOLOv8 and two-stage for Mask-RCNN) significantly influenced the outcomes.

### 4.1. Performance of Custom Models

The Mask R-CNN-based models demonstrated superior performance at the pixel level compared to the one-stage YOLOv8 models. Specifically, Mask R-CNN achieved higher mask overlap (MIoU), lower averages of False Positives (FPs) and False Negatives (FNs), and more consistent precision and recall across various confidence thresholds (CTs). For instance, the mask_rcnn_R101_FPN_3x model at a CT of 0.6 exhibited robust pixel-level segmentation. In contrast, YOLOv8 models, particularly YOLOv8n-Seg, prioritized inference speed over pixel-level precision, resulting in slightly lower MIoU but comparable performance in specific contexts.

Inference time analysis further highlighted the trade-offs between the two frameworks. The mask_rcnn_R101_FPN_3x model required an average inference time 4.4 times longer than YOLOv8n-Seg, underscoring YOLOv8’s advantage in applications requiring rapid processing. These results suggest that the choice of model depends on the balance between precision and computational efficiency required by the application.

### 4.2. Limitations and Trade-Offs Across Metrics and Severity Levels

Both frameworks faced challenges in generalizing across different metrics and severity levels. The optimal CT ranges for pixel-level evaluation (e.g., MIoU) did not align with those for RMSE or predicted vs. ground truth lesion areas, as the latter metrics are sensitive to the balance between FPs and FNs during mask generation. Similarly, RMSE, which operates on continuous values, did not directly correlate with severity level classification, which relies on discrete intervals (levels 0, I, II, and III).

Mask R-CNN’s conservative inference style, prioritizing precision over recall, led to smaller predicted lesion areas, particularly for leaves with higher severity. This conservatism caused an accumulation of errors, resulting in underestimated severity values, as evidenced by residuals, regression, and boxplot analyses. Most classification errors occurred near interval boundaries, posing a significant challenge for Mask R-CNN in severity-level classification. Conversely, YOLOv8 models exhibited greater variability in predicted lesion areas, balancing over- and underestimation, which contributed to better performance in high-severity cases. The YOLOv8n-Seg model at a CT of 0.6–0.7 outperformed Mask R-CNN in severity-level classification, despite its pixel-level precision trade-offs.

It is important to note, however, that the test dataset included samples only up to severity level III (3.5–8.0% of affected leaf area), as higher severity levels (IV to VI) were not observed in the field-collected data.

### 4.3. Implications for Agricultural Applications

The findings have significant implications for deploying these models in agricultural settings, particularly for disease monitoring in chili pepper crops. YOLOv8n-Seg’s superior performance in severity-level classification and faster inference times make it a promising candidate for real-time applications, such as automated field monitoring systems. Its ability to balance prediction errors across severity levels enhances its reliability for practical use, where rapid and accurate severity assessment is critical for timely interventions.

However, Mask R-CNN’s pixel-level precision may be preferable in applications requiring detailed lesion mapping, such as research-oriented studies or precision agriculture systems that prioritize segmentation accuracy over speed. The choice of CT is crucial in optimizing model performance, as suboptimal thresholds can exacerbate errors near severity interval boundaries. Future work should focus on developing adaptive CT selection methods and hybrid models that combine the strengths of both frameworks to improve generalization and robustness in diverse agricultural contexts.

## 5. Conclusions

In recent years, agricultural research has increasingly leveraged deep learning technologies to improve modern practices, enhance crop quality, automate processes, and promote sustainability. This study specifically undertook a comprehensive experiment to evaluate and compare the performance of two recent and widely used deep learning models—YOLOv8 and Mask R-CNN—in the segmentation and automatic calculation of disease severity in plants. The study’s particular emphasis was on Cercospora leaf spot in chili peppers. Based on the results, the following key conclusions are drawn:

1. Mask R-CNN models excelled in pixel-level metrics, with the mask_rcnn_R101_FPN_3x model achieving an MIoU of 86% and an F1-score of 92.4%. The best-performing YOLOv8 model, YOLOv8s-Seg, recorded an MIoU of 80.8% and an F1-score of 89.3%.
2. In severity-level classification, YOLOv8 models outperformed, with YOLOv8n-Seg achieving the highest F1-score of 95.1%. In comparison, the best Mask R-CNN model achieved an F1-score of 94.7% in this task.
3. Despite the superior pixel-level metrics, Mask R-CNN models showed a tendency to underestimate severity. This resulted in predicted areas generally smaller than the ground truth, leading to more significant errors in classifying higher severity levels.
4. The confidence threshold (CT) proved to be a critical performance factor. YOLOv8n-Seg, although sensitive to changes in CT, delivered the best severity classification results at its optimal threshold.
5. YOLOv8 models demonstrated significantly faster average inference times than Mask R-CNN models, with the fastest YOLOv8 model processing at 27 ms and the best Mask R-CNN model at 89 ms.
6. This study underscores the importance of carefully selecting evaluation metrics based on the specific application. The choice of metrics can significantly impact the perceived performance of the models, highlighting that the final application should guide the evaluation criteria.

## 6. Future Work

Future research could focus on fine-tuning the hyperparameters of the training process to explore potential improvements in model performance. Optimizing these parameters may lead to more accurate predictions and overall better model effectiveness. Additionally, future studies should aim to collect samples that represent higher severity levels (above severity level III) in order to more comprehensively assess the models’ generalization across the full range of severity classes. This would provide a better understanding of how the models handle varying degrees of disease severity. Moreover, researchers could evaluate the recently released YOLOv9 and YOLOv10 models, testing them against the dataset to assess pixel-level accuracy and severity classification precision. By comparing these newer models with YOLOv8, future studies can provide valuable insights into advancements in object detection and segmentation technologies and their specific applicability in tasks like evaluating Cercospora leaf spot severity in chili peppers.

## Figures and Tables

**Table 1:** Dataset general aspects.

Dataset Split	Samples n°	Lesions Annotated
Train	1040	3943
Valid	294	1131
Test	309	1208
Total	1645	6282

**Table 2:** Severity-level scale based on severity %.

Severity (%)	Level
Healthy	0
0–1.5	I
1.5–3.5	II
3.5–8.0	III
8.0–16	IV
16.3–31	V
≥50	VI

**Table 3:** mAP results from validation dataset.

Model	mAP50
mask_rcnn_R101_FPN_3x	0.894
mask_rcnn_R50_FPN_3x	0.893
mask_rcnn_R101_C4_3x	0.901
mask_rcnn_R50_C4_3x	0.897
YOLOv8n-Seg	0.925
YOLOv8s-Seg	0.915
YOLOv8m-Seg	0.909
YOLOv8l-Seg	0.906

**Table 4:** Pixel-level metrics’ evaluation.

Model	MIoU	Accuracy	Precision	Recall	F1-Score	CT	Inf. Time (ms)
mask_rcnn_R101_FPN_3x	0.860	0.999	0.942	0.907	0.924	0.3	119
mask_rcnn_R50_FPN_3x	0.848	0.999	0.957	0.881	0.918	(0.6–0.7)	89
mask_rcnn_R101_C4_3x	0.838	0.999	0.956	0.870	0.911	0.8	473
mask_rcnn_R50_C4_3x	0.840	0.999	0.942	0.883	0.911	0.9	477
YOLOv8n-Seg	0.807	0.998	0.911	0.853	0.881	0.6	27
YOLOv8s-Seg	0.808	0.998	0.905	0.882	0.893	(0.4–0.5)	34
YOLOv8m-Seg	0.805	0.998	0.906	0.876	0.891	(0.1–0.4)	41
YOLOv8l-Seg	0.805	0.998	0.906	0.878	0.892	(0.2–0.4)	62

**Table 5:** RMSE results.

Model	RMSE	CT
mask_rcnn_R101_FPN_3x	0.132	0.3
mask_rcnn_R50_FPN_3x	0.184	0.3
mask_rcnn_R101_C4_3x	0.186	0.1
mask_rcnn_R50_C4_3x	0.159	0.1
YOLOv8n-Seg	0.153	0.6
YOLOv8s-Seg	0.136	0.3
YOLOv8m-Seg	0.150	0.1
YOLOv8l-Seg	0.149	0.2

**Table 6:** Severity classification metric results.

Model	Precision	Recall	F1-Score	CT
mask_rcnn_R101_FPN_3x	0.949	0.948	0.947	0.6
mask_rcnn_R50_FPN_3x	0.928	0.926	0.924	(0.3–0.4)
mask_rcnn_R101_C4_3x	0.931	0.929	0.928	(0.1–0.2)
mask_rcnn_R50_C4_3x	0.930	0.929	0.928	(0.1)
YOLOv8n-Seg	0.952	0.951	0.951	(0.6–0.7)
YOLOv8s-Seg	0.949	0.948	0.948	(0.3–0.4)
YOLOv8m-Seg	0.946	0.945	0.948	(0.1–0.4)
YOLOv8l-Seg	0.949	0.948	0.948	(0.1)

## Data Availability

The raw data supporting the conclusions of this article will be made available by the authors on request.
